# An Infant With Seborrhoeic Dermatitis and Eczema Herpeticum Complicated by a Generalized Infection

**DOI:** 10.7759/cureus.16818

**Published:** 2021-08-01

**Authors:** Katarzyna Karpierz, Ernest P Kuchar

**Affiliations:** 1 Pediatrics with Clinical Assessment Unit, Medical University of Warsaw, Warsaw, POL

**Keywords:** bacterial superinfection, risk factors, invasive infection, life-threatening sequellae, kids, skin disease/ dermatology, local therapy, id critical care, intenssive care

## Abstract

Primary herpes simplex virus 1 (HSV-1) infection in children (beyond the neonatal period) may be asymptomatic or manifest as herpetic gingivostomatitis accompanied by fever and other symptoms. However, severe, health- and life-threatening infection is observed in rare cases, especially in at-risk patients. Children with atopic dermatitis may develop extensive eczema herpeticum (*eruptio varicelliformis Kaposi*). Herpes simplex eye infection, herpes simplex encephalitis, and disseminated (generalized) herpes infection also pose danger. We present a boy with exacerbated infantile seborrhoeic dermatitis (ISD) and eczema herpeticum complicated by streptococcal sepsis. HSV transmission should be limited if possible by avoiding direct contact with those who recently developed lesions. Communication with parents and explaining how to properly care for the skin in a child with skin diseases that disturb its barrier function protecting against external factors is particularly important. Also, parents should be informed that "red flag" symptoms in a child should be an indication for a pediatric consultation. In the event of infection, the duration of symptoms can be reduced by promptly initiated acyclovir therapy.

## Introduction

Primary herpes simplex virus 1 (HSV-1) infections in children, beyond the neonatal period, may be asymptomatic or manifest as gingivostomatitis accompanied by fever, and, often, dehydration, sporadically presenting as severe forms of disseminated herpes infections, herpes encephalitis, or herpetic eye disease with keratitis or retinal involvement [1].

In young children, the primary infection most typically manifests as herpetic gingivostomatitis, which develops in about one-fourth of children aged six months to five years [2]. The group at risk of severe infection includes newborns, immunocompromised children, and patients with chronic, extensive skin diseases (such as seborrhoeic or atopic dermatitis). In the latter group, the infection usually takes the form of eczema herpeticum (*eruptio varicelliformis Kaposi*), i.e. disseminated vesicular eruptions with an aggressive course. Herpes encephalitis is a severe disease of the central nervous system (CNS) associated with a high risk of complications and mortality [1].

The incubation period for herpes is two to 14 days (mean of four days). The infection occurs through contact with skin lesions or saliva of a symptomatic patient or, importantly, an asymptomatic person who, after being infected, periodically sheds HSV throughout their life, posing a potential threat to non-immune exposed individuals. In the case of primary infection, autoinoculation (self-infection), i.e. transmission of the virus to other parts of the body by directly touching the lesions, is also possible. Such spread of the virus during a relapse is limited by serum antibodies [1].

Acyclovir and its derivatives are currently available agents that inhibit HSV replication. The dosage, route of administration (IV or oral), and length of treatment depend on the form of the disease and other comorbidities [3].

We present a boy with exacerbated infantile seborrheic dermatitis and eczema herpeticum complicated by *Streptococcal sepsis*.

## Case presentation

**Figure 1:**
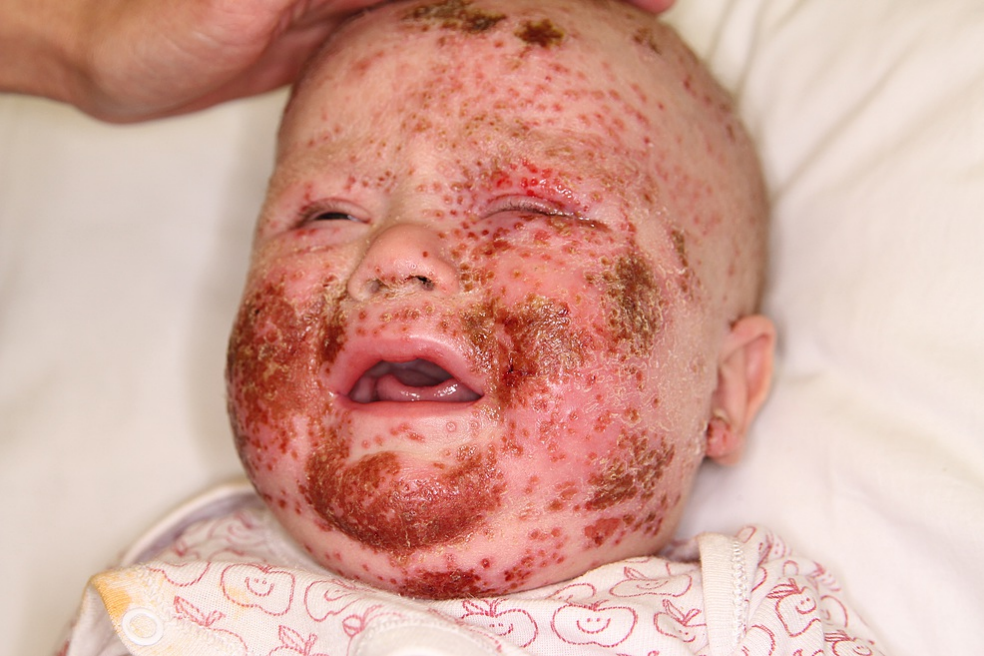
Skin lesions on the face of the boy with the left eye involvement. Notice the left eye involvement, vesicles, erosions, and confluent crusts on chicks and chin

A seven-month-old boy with severe infantile seborrhoeic dermatitis (ISD) was admitted to the Department of Paediatrics with the Observation Unit due to a suspected systemic infection. The boy’s parents noticed an increased severity of seborrhoeic skin lesions two days before admission. He developed a fever of up to 38.5°C every six hours, with a further increase in body temperature after one day (episodes of fever up to 40°C every four hours). There was a striking, significant, and rapid progression of skin lesions, with extensive, oozing skin erosions, vesicles, and crusts. The child was less active, with periodically increased somnolence or irritability. When irritated, the boy tilted his head back and presented with eye deviation. The parents negated other symptoms in the child as neck stiffness, photophobia, hyperesthesia, nausea, or vomiting. The boy was reported to the hospital due to a deteriorating general condition. Furthermore, it was reported in medical history that both boy’s parents and his sister suffer from recurrent labial herpes, although with no recent eruptions (Figure 1).

The child’s medical history revealed that the boy was generally healthy with ISD diagnosed at the age of two months and that the present exacerbation was so far the most severe one. He received vaccinations up to the age of six weeks. The remaining vaccinations were postponed by a dermatologist (as reported by the mother). Perinatal and family history was unremarkable.

The child was in moderate overall condition on admission. He was irritable and prone to crying but with normal vital parameters. Physical examination revealed the following abnormalities: multiple vesicles and extensive dark honey-colored crusts covering a large skin surface on the face and dorsal hands, with a tendency to ooze and slightly bleed. Similar yet less severe, lesions were observed on the lower limbs. Furthermore, significant edema of the left eyelids with purulent discharge and multiple vesicles on the eyelids and forehead were observed. Additionally, a raised, pulsating fontanelle and rhythmic tongue clicking, which were not previously observed by the parents, were noticeable.

Laboratory tests showed moderately elevated inflammatory markers (C-reactive protein, CRP - 1.9 mg/dL [N: <1];), increased leukocytosis up to 22000/uL with a neutrophil count of 61%; other than that the blood count was normal; the electrolytes, arterial blood gas, renal function markers, and coagulation profile were within the normal range. The urinalysis showed no signs of infection. Two blood cultures and some cultures from skin lesions were collected. Herpes infection, skin and soft tissue infection complicated by sepsis were suspected. 

On admission, broad-spectrum antibiotic therapy was used as in sepsis (ceftriaxone and vancomycin); acyclovir was included in the treatment accordingly to presumed diagnosis. Furthermore, the child was hydrated and received analgesic and antipruritic treatment. The next day, the child was very restless, with head tilted back, pulsating fontanelle, and periodical eyeball deviation. A decision was made to perform a lumbar puncture to assess CNS involvement; however, normal cerebrospinal fluid (CSF) and negative CSF viral panel for HSV obtained two days later excluded neuroinfection. During the child’s hospital stay, attention was drawn to herpetic eruptions present on the nipple of his mother. Samples were additionally collected from the nipple for HSV polymerase chain reaction (PCR) testing to confirm the diagnosis and were positive. Methicillin-sensitive *Staphylococcus aureus* and *Streptococcus pyogenes* were isolated from skin lesion cultures, and *S. pyogenes* was isolated from blood cultures. After obtaining antibiograms of the isolated microorganisms on the fourth day of the therapy, the treatment was changed to cloxacillin monotherapy. Intravenous antibiotic and acyclovir treatments were continued until day 8. Then, the treatment was changed to a first-generation oral cephalosporin (up to 14 days in total) and oral acyclovir due to difficulties in maintaining IV access. From the third day of hospital stay, swelling of the abdomen and limbs was observed; laboratory work-up showed reduced albumins (without proteinuria), but no IV albumin was given. In the three days that followed, the swelling subsided and the child's condition gradually improved. Ultrasound (US) of the dorsal parts of the swollen hands showed no signs of an abscess. A consulting ophthalmologist found no ocular involvement on the second day of the hospital stay. Rapid fever resolution and gradual regression of superinfected facial skin lesions were observed. Due to an abnormal transfontanellar US (the changes probably occurred earlier - slight dilatation of the lateral ventricles and the third ventricle, features of the thalamic vasculopathy), an electroencephalogram (EEG) (normal) was performed, and further outpatient neurological follow-up was recommended.

The child was ultimately diagnosed with Streptococcal sepsis originating from superinfected skin lesions in dermatitis (staphylococcal and streptococcal) in the course of eczema herpeticum. The boy was discharged home in good general condition.

## Discussion

Special vigilance and rational, proper management are needed in a patient with suspected systemic infection. In such cases, the collection of swabs and cultures before the administration of the first dose of an antibiotic is a key element of the initial management. In our patient, two blood cultures and three swabs were collected -- from the left conjunctival sac and facial skin lesions. After stabilization of the patient's condition, the results of the cultures allowed for a safe de-escalation of the applied empirical therapy, which was immediately switched to targeted treatment. 

An increased transaminase activity characterizes generalized HSV infection and normal transaminase levels are an argument against generalized HSV infection. A lumbar puncture with a general assessment of the CSF should be performed in each patient with herpes and suspected CNS involvement to verify the presence of HSV genetic material in the fluid. Apart from the obvious prognostic assessment, it has a significant impact on the duration and route of acyclovir treatment. The treatment of CNS infections must be IV and longer than the topical approaches. In our patient, disturbing symptoms included pulsating fontanelle, smacking the tongue, eyeball deviations, and a tendency to periodically arch his body back. Due to the completely normal CSF containing no viral genetic material, the symptoms (which resolved in the following days) were most likely caused by systemic infection. Localization of eczema herpeticum lesions near the eye required ophthalmological consultation to exclude corneal involvement, which would also require prolonged IV therapy. The choice of other diagnostic tools depends on the patient's condition. In our patient, non-invasive imaging in the form of transfontanellar US was performed, followed by EEG due to minor abnormalities found in the US and the planned future outpatient neurological follow-up, to facilitate the diagnosis ordered by the neurologist. Due to the asymmetric edema of the hands affected by skin lesions, a US evaluation was needed to verify the possible presence of abscesses that would require drainage. Recommendations at discharge should be also mentioned: in addition to general pediatric recommendations, outpatient neurological follow-up as well as dermatological care, and proper management of skin lesions, the parents were informed on the need to arrange for any missing vaccinations in the child. Parents should be always informed that a chronic skin disease is not an indication to postpone preventive vaccinations.

## Conclusions

Infants with atopic dermatitis and other chronic skin diseases are at risk of developing bacterial superinfections and a severe primary HSV infection.
